# Fast Mass Microscopy: Mass Spectrometry Imaging of a Gigapixel Image in 34 Minutes

**DOI:** 10.1021/acs.analchem.2c02870

**Published:** 2022-10-12

**Authors:** Aljoscha Körber, Joel D. Keelor, Britt S. R. Claes, Ron M. A. Heeren, Ian G. M. Anthony

**Affiliations:** † The Maastricht MultiModal Molecular Imaging Institute (M4i), Division of Imaging Mass Spectrometry, Maastricht University, Universiteitssingel 50, Maastricht 6229 ER, The Netherlands; ‡ Amsterdam Scientific Instruments B.V. (ASI), Science Park 106, Amsterdam 1098 XG, The Netherlands

## Abstract

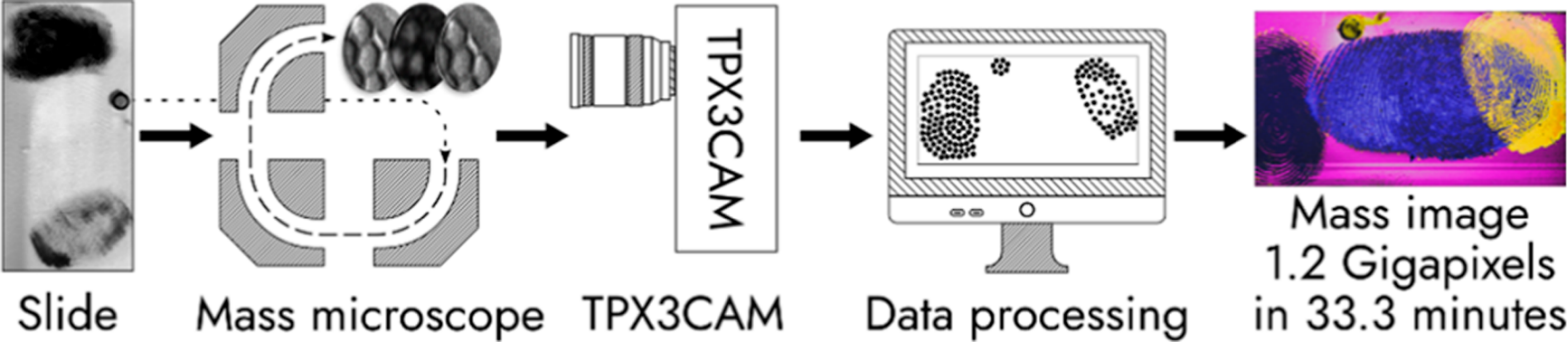

Mass spectrometry imaging (MSI) maps the spatial distributions of chemicals on surfaces. MSI requires improvements in throughput and spatial resolution, and often one is compromised for the other. In microprobe-mode MSI, improvements in spatial resolution increase the imaging time quadratically, thus limiting the use of high spatial resolution MSI for large areas or sample cohorts and time-sensitive measurements. Here, we bypass this quadratic relationship by combining a Timepix3 detector with a continuously sampling secondary ion mass spectrometry mass microscope. By reconstructing the data into large-field mass images, this new method, fast mass microscopy, enables orders of magnitude higher throughput than conventional MSI albeit yet at lower mass resolution. We acquired submicron, gigapixel images of fingerprints and rat tissue at acquisition speeds of 600,000 and 15,500 pixels s^–1^, respectively. For the first image, a comparable microprobe-mode measurement would take more than 2 months, whereas our approach took 33.3 min.

## Introduction

Chemical surface imaging has enabled breakthroughs in fields from material sciences^1−3^ to biology.^4−7^ Mass spectrometry imaging (MSI) is a chemical surface imaging technique that offers the highest chemical information density of frequently used surface-imaging techniques.^5,6,8^

Most MSI experiments use the microprobe-mode, wherein a laser or ion beam is scanned pixel-by-pixel over a surface. Each pixel corresponds to a single mass spectrum. The most common laser and ion beam-based MSI methods are matrix-assisted laser desorption/ionization (MALDI) and secondary ion mass spectrometry (SIMS), respectively.^5^ Microprobe-mode MSI is time-consuming and can require hours or even days of imaging when small pixel sizes of less than 10 μm are used.^9^ Small pixels are necessary to resolve small features on the sample, for instance, a tumor cell surrounded by healthy tissue.^10^ Thus, decreasing pixel size is a major focus of MSI advancements^11−13^ and has enabled MSI to become widely used for single cell metabolomics,^14,15^ drug development,^16,17^ and pathology.^4,18^ However, a linear decrease in microprobe-mode pixel size necessitates a quadratic increase in the number of pixels needed to scan the same spatial area. Thus, every improvement in reducing the microprobe-mode pixel size leads to lower throughput and hence to smaller imaged areas or longer acquisition times. Researchers need higher throughput MSI and have expressed this need multiple times over the last decade,^14,16,18−20^ High throughput is especially needed for imaging large numbers of samples^7,18^ and for time-critical applications, such as intraoperative cancer diagnosis.^21^

Efforts to increase MSI throughput have mostly focused on microprobe-mode MALDI but are analogously applicable to SIMS.^9,22−25^ Throughput improvements have been achieved using the following: higher repetition rate primary beams,^22^ continuous sample scanning,^23^ and rastering the primary beam instead of moving the sample, to scan the sample.^24,25^ An alternative to the microprobe-mode is microscope-mode MSI (mass microscopy), in which under high vacuum conditions, an ion image is extracted from the sample, preserved during time-of-flight (TOF) mass analysis, and magnified onto a spatially sensitive detector.^5,26,27^ In mass microscopy, the spatial resolving power is ion-diffraction limited and depends solely on the quality of the detector and ion-optics.^26^ Mass microscopy allows many mass spectra to be acquired in parallel, rather than sequentially. The throughput of microscope-mode MSI is thus independent of the pixel size of the resulting mass image. This independence allows much shorter measurement times when compared to microprobe-mode MSI, especially at less than 10 μm pixel sizes. No mass-resolved, high-throughput mass microscopy studies have yet been conducted, although non-mass resolved stigmatic ion imaging (a technique related to mass microscopy) has shown potential for increased throughput compared to microprobe MSI.^28^

Herein, we report the development of a continuous-acquisition, high-throughput, mass microscopy method that uses a previously described instrument^27,29^ in conjunction with a TPX3CAM, a Timepix3-based hybrid pixel camera detector with single ion sensitivity,^30^ to enable spatially and mass-resolved ion detection. Using this fast mass microscope setup with metal-assisted SIMS, we acquired a 1.2 gigapixel mass image of a 42 × 23.5 mm^2^ area (roughly the area of half a microscope slide) with an effective pixel size of 900 nm in 33.3 min. We also collected images of murine and human tissue sections to demonstrate the applicability of fast mass microscopy to biological samples. Last, we compare mass resolving power of our method with state-of-the-art microprobe-mode MSI and discuss potential improvements to reach parity.

## Materials and Methods

### Instrumentation

**Figure 1:**
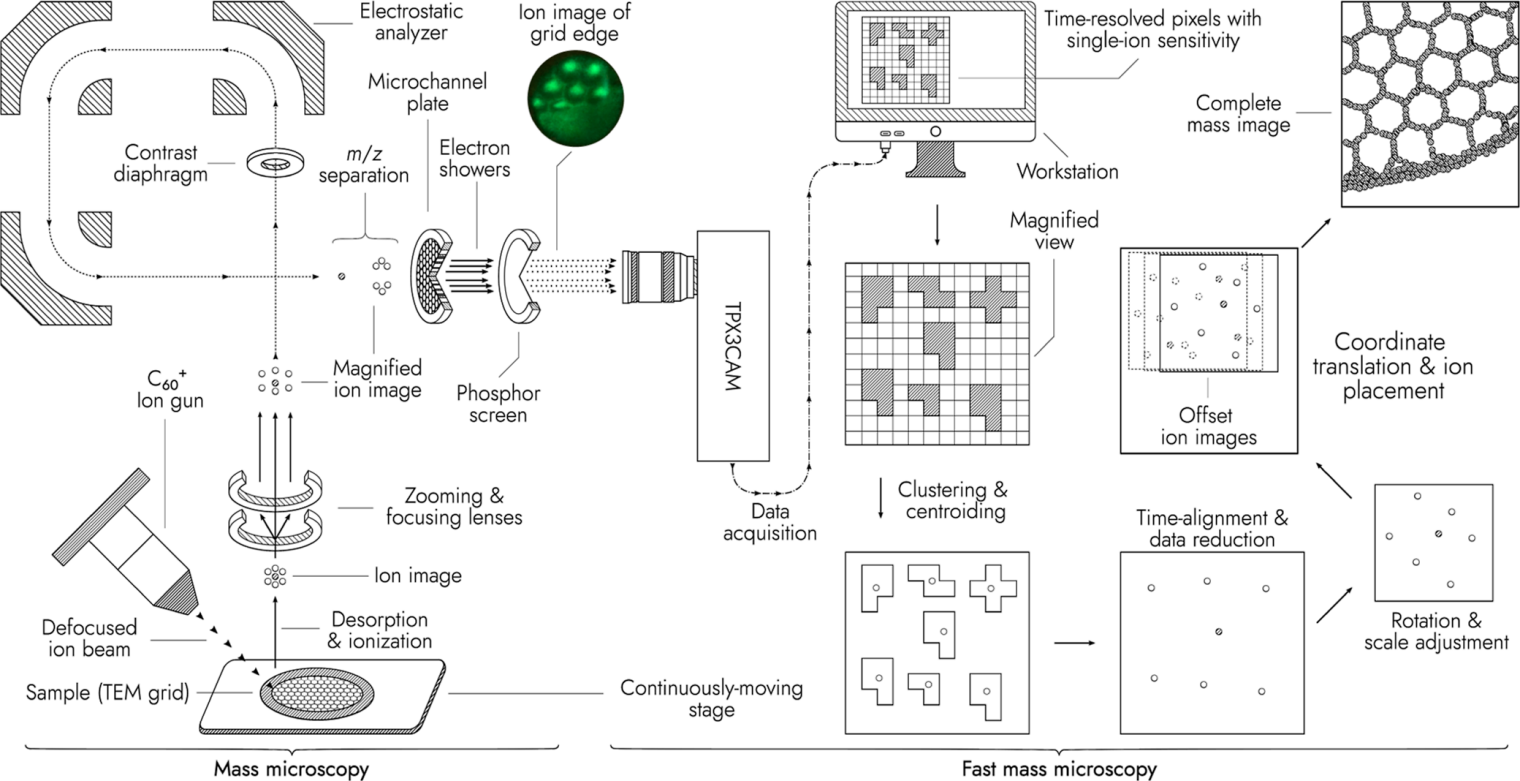
Scheme of the mass microscope modified with a TPX3CAM detector. A defocused C_60_^+^ ion beam irradiates a sample on a quickly and continuously moving stage, generating an ion image. The ion image is extracted into a TOF analyzer and magnified. The mass-separated ion image is projected onto a microchannel plate (MCP), producing electron showers. The electrons are converted into photons by a P43 phosphor screen. The photons are recorded with the TPX3CAM. The data are clustered and time-aligned to sets of single-ion impact coordinates. Ion coordinates are translated to sample stage coordinates and a larger ion image of the stage-scanned sample surface is constructed. A photo of the phosphor screen (top-left corner) shows the distribution of surface ions when the fullerene ion beam irradiates the edge of a TEM grid.

The BioTrift, an instrument based on the TRIFT II mass microscope [Physical Electronics, Inc. (PHI) Chanhassen, MN, USA]^31^ and equipped with a C_60_ ion beam (IOG C60-20S, Ionoptika, Chandler’s Ford, UK),^27^ was modified by mounting a Timepix3 ASIC-based camera (TPX3CAM, Amsterdam Scientific Instruments, Amsterdam, NL) that replaced the original CCD-based camera that collected photons emitted by the phosphor screen, which in turn observed a MCP detector with 12 μm pores and 15 μm pitch (Figures 1 and S1). The TPX3CAM was fitted with an adjustable TV zoom lens (Zoom 7000 Navitar Inc., Japan) and was mounted using a custom bracket designed and machined at M4i (Maastricht, NL). The data from the TPX3CAM were recorded using the SoPhy software package (SoPhy 1.6.3, ASI) in the 10 GBPS continuous mode.

### Custom Stage and Trigger Control Software

A Python (CPython, 3.8.0, Python Software Foundation, DE, USA) program using the Kivy (version 2.0.0) and pySerial (version 3.5.0) libraries was written to supply UART control commands to both the BioTrift mass microscope’s stage and a microcontroller (STM32F411, Estardyn online store) with firmware written using the Arduino Software Platform (Arduino, Somerville, MA, USA). The stage was continuously moved in a serpentine pattern during each imaging row to allow for rapid image generation. The microcontroller synchronized signals from the BioTrift mass microscope (generated using the WinCadence version 5.2.0.1 control software, ULVAC PHI) to both the C_60_ ion gun and the TPX3CAM detector. Two DG535 digital delay generators (Stanford Research Systems, Sunnyvale, CA, USA) provided time alignment and relaying of the signals between the microcontroller and the C_60_ ion gun and TPX3CAM. A triggering delay of approximately 300 ms at the end of each imaging row provided information for row-end determination.

### Ion-Optical Alignment

For every newly loaded sample, a real-time, continuous, stigmatic ion “video” of a portion of the 300 mesh transmission electron microscopy (TEM) grid was initiated in SoPhy. The immersion lens, transfer lens, MCP gain, phosphor screen, and C_60_ ion gun parameters were adjusted to optimize the focus, brightness, and field-of-view of the acquired stigmatic ion “video”. The resulting ion optical magnification was approximately 70, meaning that 1 μm^2^ on the sample was observed by 4900 μm^2^ of the MCP detector. The TV zoom lens of the TPX3CAM was adjusted as needed. Ion-optical alignment took approximately 2 min.

### Mass Spectrometry Imaging

For all images collected using the BioTrift mass microscope, the C_60_ ion gun and the TPX3CAM were triggered externally by the BioTrift’s original software. The C_60_ ion beam aperture was set to 1 mm, defocused to fill the entire field of view of approximately 320 μm in diameter, and optimized to produce the highest continuous ion beam current (0.5–0.8 nA) at a source temperature of 410 °C. The second-largest BioTrift contrast diaphragm aperture was used for all images unless stated otherwise. The contrast diaphragm reduces the energy spread of the ion images and smaller apertures allow for crisper images at the cost of decreased ion transmission.

### Chemicals and Materials

Ethanol (HPLC grade), *n*-hexane (HPLC grade), and xylene (AR grade) were purchased from Biosolve (Valkenswaard, NL). Hematoxylin and Entellan were purchased from Merck (Darmstadt, DE). Eosin-Y was purchased from J.T. Baker (Center Valley, USA). “Pilot Blue-black” ink was purchased from Pilot Corporation (Tokyo, JP). Yellow “Hype” highlighter (Staples, Framingham, USA) and black “edding 3000” permanent markers (edding, Ahrensburg, Germany) were purchased from a local stationery story (Maastricht, NL). Thin bar, 3.05 mm, copper TEM grids with mesh sizes of 300 (hexagonal) and 2000 (square) were purchased from Agar Scientific, Ltd (Stansted, Essex, UK). Conductive indium tin oxide (ITO)-coated glass slides were purchased from Delta Technologies (Loveland, CO, USA) and were cleaned with hexane and ethanol. Mouse kidney, rat brain, and human intestine were obtained from The Johns Hopkins University School of Medicine and Maastricht University, respectively. The Institutional Animal Care and Use Committees granted ethical approval under A3272-01 and DEC 2014-085 for the mouse and rat tissues, respectively. Human intestinal tissue was a granted ethical approval under METC 06-3-044. All organs were snap-frozen in liquid nitrogen. Cryo-sections (10 μm thickness) were prepared with a cryostat (Leica Biosystems, Wetzlar, DE) at −20 °C, thaw-mounted on ITO-coated glass slides and stored at −80 °C.

### Sample Preparation and Measurement

A single 300 mesh TEM grid was adhered with a highlighter marker to an unobtrusive location near the center of each sample slide. After application of the TEM grid, each slide was coated with 1.0 nm of gold using a high-resolution sputter coater (SC7640, originally Polaron Ltd, now Quorum Technologies, Laughton, UK) and then loaded into the BioTrift mass microscope. The gold sputter-coating step was completed in less than 3 min and served to enhance the signal.^5^

Fingerprints were collected from an author of this paper. For collection, the author washed both hands with soap for 60 s, rinsed with tap water, and dried with a paper towel. After washing, the author’s (1) right index finger was inked with a black permanent marker, (2) right little finger was inked with a pen containing blue-black ink, and (3) right thumb was groomed on the author’s forehead and nose. Immediately after each finger was prepared, the finger was placed firmly on the same ITO-coated glass slide and rolled, producing a slide with three total fingerprints.

The slide was imaged with settings of 2000 shots per area, emphasizing speed, row-overlap of 33%, C_60_ pulse time of 300 ns, and an *m*/*z* range from 0.5 to 200 Da. The imaging area was 42.0 × 23.5 mm^2^ with an acquisition time of 00:33:19. The ion dose was equal to or less than 7.9 × 10^9^ cm^2^.

The combined time for the steps of sample preparation (less than 5 min), sample transfer (less than 10 min), instrument calibration and software setup (less than 5 min), imaging (33.3 min), and conversion to mass image views (≈30 min) was less than 85 min.

The spatial resolving power test of the BioTrift was conducted by imaging a 1.0 × 1.0 mm^2^ area of a 2000 mesh grid with the third largest contrast diaphragm.

For depth-of-field resolution testing, a deformed 300 mesh copper TEM grid was placed on top of another 300 mesh TEM grid on an ITO slide. A 4 × 5.5 mm^2^ area was imaged.

Slides containing tissue sections were transferred from the −80 °C freezer to a transport chamber containing silica-gel desiccant to minimize condensation. After transport, each slide was placed in a desiccator for 15 min to remove moisture and warm to room temperature.

Rat brain and mouse kidney images were with settings of 50,000 shots per area, emphasizing signal, row-overlaps of 75%, C_60_ pulse times of 150 ns, and *m*/*z* ranges from 0.5 to 500 Da. Human intestine was imaged with 200,000 shots per area under otherwise identical experimental conditions. Brain, kidney, and intestine and kidney imaging areas were 18.0 × 16.5, 6.0 × 6.0, and 10.4 × 11.8 mm^2^ with acquisition times of 21:03:30, 02:33:40, and 2:23:24, respectively. The ion dose for both experiments was equal to or less than 3.4 × 10^11^ cm^2^. The ion dose per unit area for the tissue is ∼20 times below the static limit.^5^

After imaging with the BioTrift mass microscope, the rat brain sample was transferred to a nanoTOF II (PHI) imaging mass spectrometer and a 2.5 × 2.5 mm^2^ region was imaged using a C_60_ ion beam (IOG C60-20S, Ionoptika, Ltd) with a raster pixel size of 2 μm and an acquisition rate of 200 pixels s^–1^ in the mosaic microprobe imaging mode.

### Hematoxylin and Eosin Staining

Consecutive mouse kidney and human intestine sections were stained to compare hematoxylin and eosin (H&E) images with fast mass microscopy images. The prepared slides and tissues were immersed in the following (in order): 70% ethanol for 180 s, MilliQ water for 180 s, hematoxylin for 180 s, running tap water for 180 s, eosin for 30 s, running tap water for 60 s, ethanol for 60 s, and xylene for 30 s. After staining, slides were covered with a coverslip using Entellan mounting medium and then imaged with a digital pathology slide scanner (Aperio CS2, Leica Biosystems, Wetzlar, DE) at 20× magnification.

### Data Processing

All data processing was completed on a single HP EliteDesk 800 G5 computer with a 3.6 GHz i7-9700K Intel Core 8 processor CPU, 32 GB 3.6 GHz RAM, 472 GB NVMe SSD. A set of scripts written in Rust^32^ were used to convert each TPX3CAM-acquired image (.tpx3 file format) into a clustered, centroided, and compressed file (.tpx3c file format) and then to convert that file into a series of mass images (.png file format) or imaging mass spectrometry data files (.imzml file format, see the Supporting Information for a detailed description of the file conversion).

All rate-limiting computational steps for generating the series of mass images were parallelized (using the Rayon parallelism library, version 1.5). In general, image conversion required comparable or less time than imaging. For example, complete processing (to .tpx3c and then to .png images) of the fingerprints image (a 65.0 GB file in .tpx3 file format) required approximately 30 min of elapsed real time. Complete processing of the brain image (a 477.8 GB file in .tpx3 file format) required approximately 5 h of elapsed real time. As the image processing has been parallelized, processing on, for example, cluster computers could reduce this time substantially as could using faster solid state drives or processing during image acquisition.

## Results and Discussion

**Figure 2:**
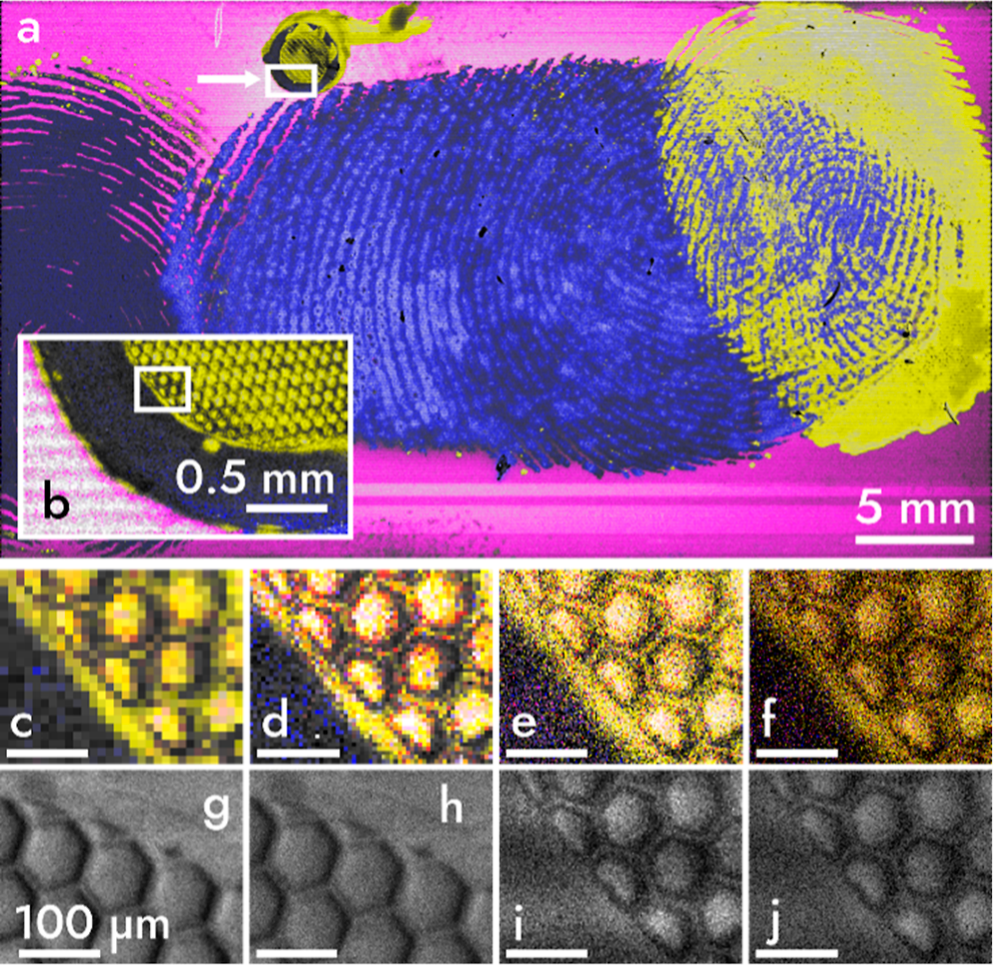
Mass image of a 42 × 23.5 mm^2^ area recorded in 33.3 min (a) A false-color mass image of three fingerprints and a TEM grid on an ITO slide with blue, magenta, and yellow colors mapped to mass-to-charge values (*m*/*z*) of 55, 115, and 23 *m*/*z*, which are tentatively assigned to either C_4_H_7_^+^ or C_3_H_3_O^+^, In^+^, and Na^+^, respectively.^33^ A white arrow in (a) points to a box indicating an intermediate zoom (b) of the TEM grid, displayed at a pixel size of 10 μm. A white box in (b) highlights the region of the grid used for magnification in (c–f) that are viewed with pixel sizes of 10 (c), 5 (d), 2 (e), and 0.9 (f) μm. Total ion count (TIC) images (g–j) are shown in gray at pixel sizes of 2 (g,i) and 0.9 (h,j) μm. Images (g,h) are of an identically sized grid imaged at slower speed and show increased ion counts. Viewed at a pixel size of 0.9 μm, the image is >1.2 gigapixels.

Figure 2a shows a view of the 42 × 23.5 mm^2^ area containing a set of fingerprints and a TEM grid was imaged with a mass microscope in 33.3 min. This speed is possible due to the addition of a TPX3CAM that records individual ion impacts with a time resolution of 1.56 ns.^30^

In contrast to microprobe-mode MSI, the pixel size of images generated with fast mass microscopy is independent of spatial resolving power and ion or laser beam size. Instead, the pixel size is chosen by the viewer who can zoom the image seamlessly (see video S1). Viewing at larger pixel sizes can enhance image contrast (Figure 2b–d), whereas smaller pixel sizes improve spatial detail (Figure 2e–j). A method for selecting a “good” pixel size is by referencing the spatial resolving power of the instrument. On our setup, the spatial resolving power was measured to be at least 3.4 μm with MALDI and 2.5 μm with SIMS (Figure S2).^29,34^ The Nyquist–Shannon sampling theorem requires a pixel size of 2.5 μm/2.8 ≈ 0.9 μm to avoid undersampling.^35^ In some images, smaller pixel sizes of 0.5 μm can allow better observation of fine details (Figure S2). A drawback of submicron pixel sizes is that low-abundance mass images may contain pixels with few or no ion hits. This drawback can be overcome by increasing the number of observed ions per area. For example, by using longer acquisition times, a brighter primary ion source, higher primary acceleration voltage,^36^ or by selecting a primary ion beam with higher ionization efficiency. Ion counts could also be improved by substituting the primary ion beam with a laser for high-throughput, high spatial resolution MALDI.^26,29^ Unlike SIMS, thousands of ions are generated with each laser shot. Thus, fast mass microscopy with MALDI may allow even higher throughput and sensitivity than with SIMS, especially for larger, biologically relevant molecules, such as peptides and proteins. When viewed at a pixel size of 900 nm (Figure 2f), the total image (Figure 2a) has >1.2 billion pixels corresponding to an acquisition rate of 600,000 pixels s^–1^ or 0.49 mm^2^ s^–1^. In comparison, commercial microprobe-mode TOF–SIMS (see Figures S3 and S4) achieved a maximum speed of 200 pixels s^–1^ or 0.80 × 10^–3^ mm^2^ s^–1^ at 2 μm pixel size. Such a speed introduced image artifacts not present in fast mass microscopy. For comparison with MALDI, the fastest top-of-the-line MALDI instrument operates at 50 pixels s^–1^ or 1.25 × 10^–3^ mm^2^ s^–1^ at 5 μm pixel size.^25^ Using these numbers, fast mass microscopy is at least 2–3 orders of magnitude faster than microprobe MSI techniques while also achieving high spatial resolution.

**Figure 3:**
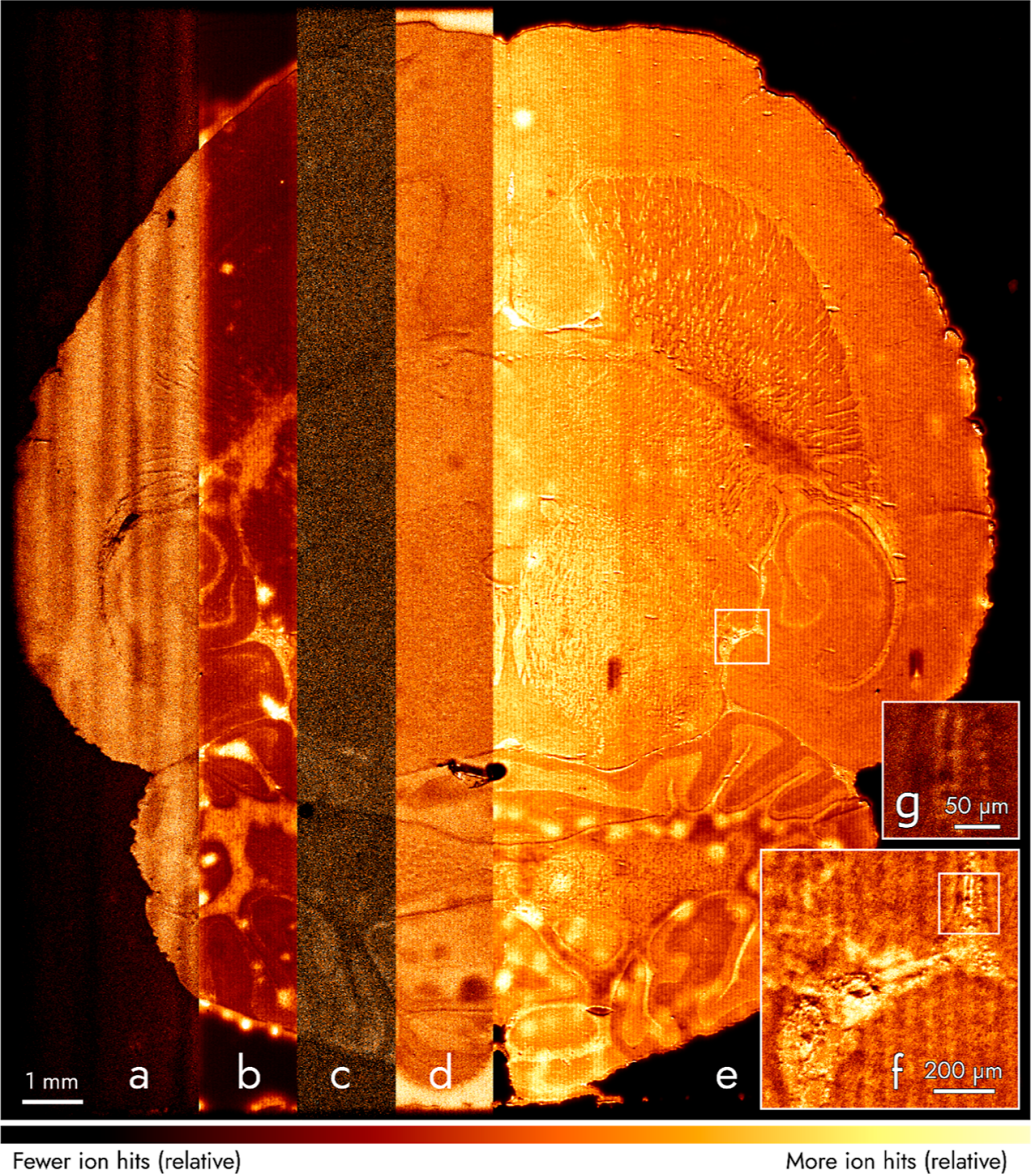
Mass image of a 16.5 × 18 mm^2^ area of a rat brain section. Areas (a–e) correspond to *m*/*z* values attributed to cholesterol (summed ion hits for M^+^ at *m*/*z* 386 and [M–H_2_O + H]^+^ at 369, respectively), Na^+^ (*m*/*z* 23), monoacylglycerides and their fragments (*m*/*z* 338 summed with 354 and 361), C_7_H_7_^+^ (*m*/*z* 91), and K^+^ (*m*/*z* 39), respectively, and are viewed with a pixel size of 5 μm. Regions in white boxes in (e,f) are magnified in (f,g) with pixel sizes of 2 and 0.5 μm, respectively. Each area (a–g) was normalized to the most intense pixel within that area. Viewed at a pixel size of 0.5 μm, the image is ≈1.2 gigapixels.

We imaged a section of rat brain at an intentionally slow speed of 3.9 × 10^–3^ mm^2^ s^–1^ (15,500 pixels s^–1^ at 0.5 μm pixels) to observe higher contrast for less abundant mass signals (Figures 3 and S3–S5). When viewed at a pixel size of 0.5 μm, the image is approximately 1.2 gigapixel large. Although imaged slower than Figure 2, the imaging rate for Figure 3 was ≈5 (measured in mm^2^ s^–1^) or ≈75 (measured in pixels s^–1^) times faster than microprobe TOF–SIMS MSI. Figure 3 shows mass images we attribute to both organic molecules, such as cholesterol (Figure 3a), and inorganic elements, such as sodium (Figure 3b). Figure 3c is a combination of mass channels, which are attributed to monoacylglycerides and their corresponding fragments. The ion count in Figure 3c is low compared to other ion images shown. Viewing at larger pixel sizes improves contrast and allows visualizing the localization of molecules as heavy as 674 Da (see Figure S5). We expect future work to allow us to increase this “useful mass range” to image, for example, phospholipids by enhancing mass resolution and ionization efficiency. Figure 3d depicts the distribution of tropylium (C_7_H_7_^+^), a fragment originating from molecules with toluene groups. Tropylium outside the tissue is attributed to the contamination of the slide or the solvents with which the slide was washed prior to mounting the tissue. Potassium (Figure 3e–g) was the most abundant mass signal and shows high contrast of the brain anatomy, even in regions with small structural features displayed at a small pixel size of 0.5 μm (Figure 3g). Figure 3 has two artifacts. First, vertical stripes visible in Figure 3a are caused by an instability of one of the ion optics the TRIFT II mass microscope. Second, localized “bright” spots observed in Figure 3b,e are attributed to water condensation on the brain tissue that occurred upon taking the tissue out of the −80 °C freezer. Following minor optimization of the sample preparation routine, these artifacts do not occur anymore in following measurements (Figures S6 and S7).

The ion signal was stable without noticeable drifts throughout this measurement (≈21.06 h) and while imaging mouse kidney (≈2.56 h) and human intestine (≈2.39 h) with similar imaging settings (Figures S6 and S7). The high primary ion dose resulted in more ion counts per pixel and better contrast without exceeding the SIMS static limit.

An advantage of fast mass microscopy, other than high throughput and high spatial resolution, is the finding that imaging surfaces with topological variations of at least 132 μm in height does not require any adjustment or compensation (Figure S8). This finding is in contrast to most microprobe-MSI approaches that are highly sensitive to changes in surface height and require tedious refocusing. This is advantageous for imaging large surface areas, as many have topology varying by more than tens of micrometers.

A current limitation of fast mass microscopy compared to microprobe-mode MSI is that the *m*/*z* resolution measured at full width at half-maximum is ≈100 at 395 *m*/*z* (Figure S4). This is neither caused by the mass microscope nor the Timepix3,^27,30^ but by 150 ns broad primary and therefore also secondary ion pulses as well as by slow rise and decay times of the phosphor screen. Future experimental work will be dedicated to reducing the ion pulse width by either combining fast mass microscopy with primary ion bunching or by switching to MALDI. These measures paired with replacing the current phosphor screen with a faster unit or direct coupling of the Timepix3 sensor to the MCP could improve mass resolution by more than an order of magnitude.

## Conclusions

MSI is generally a low-throughput method, which limits its translation into applications requiring high spatial resolution images of large sample cohorts or time-sensitive measurements. In this work, this challenge is bypassed by using high-throughput, continuous-acquisition mass microscopy instead of microprobe MSI. We achieve at least 2–3 orders of magnitude higher throughput than microprobe-mode MSI, while simultaneously achieving a high spatial resolving power of at least 2.5 μm. We believe that, after more instrumental and algorithmic development, fast mass microscopy and its future advancements will enable MSI to find more use in inorganic as well as biological and clinical surface imaging.

## Supporting Information Available

Descriptions of the experimental and the data processing workflows, custom mounting bracket, imaging data of a 12.5 μm pitch copper TEM grid, comparison of fast mass microscopy with microprobe-mode results, comparison of average mass spectra in microscope- and microprobe-mode MSI, selected ion images of the microscope-mode rat brain scan, H&E-stained light microscopy and MSI images of serial mouse kidney sections, H&E-stained light microscopy and false-color MSI images of serial human intestine sections, and TIC MSI image of two overlaying TEM grids on an ITO slide acquired using fast mass microscopy (PDF)
Seamless zooming (mpg)

## Supplementary Material

ac2c02870_si_001.pdf

ac2c02870_si_002.mpg
