# Correction to “Fast Mass Microscopy: Mass Spectrometry Imaging of a Gigapixel Image in 34 Minutes”

**DOI:** 10.1021/acs.analchem.4c00145

**Published:** 2024-02-07

**Authors:** Aljoscha Körber, Joel D. Keelor, Britt S. R. Claes, Ron M. A. Heeren, Ian G. M. Anthony

The authors note that the description of the used phosphor screen in the caption of Figure 1 is wrong. We used a P20 phosphor screen, not a P43 screen as indicated in the original manuscript. The rise time of a P43 screen is too slow to be detected with the TPX3CAM. Figure 1 with the corrected caption is depicted below.

**Figure 1:**
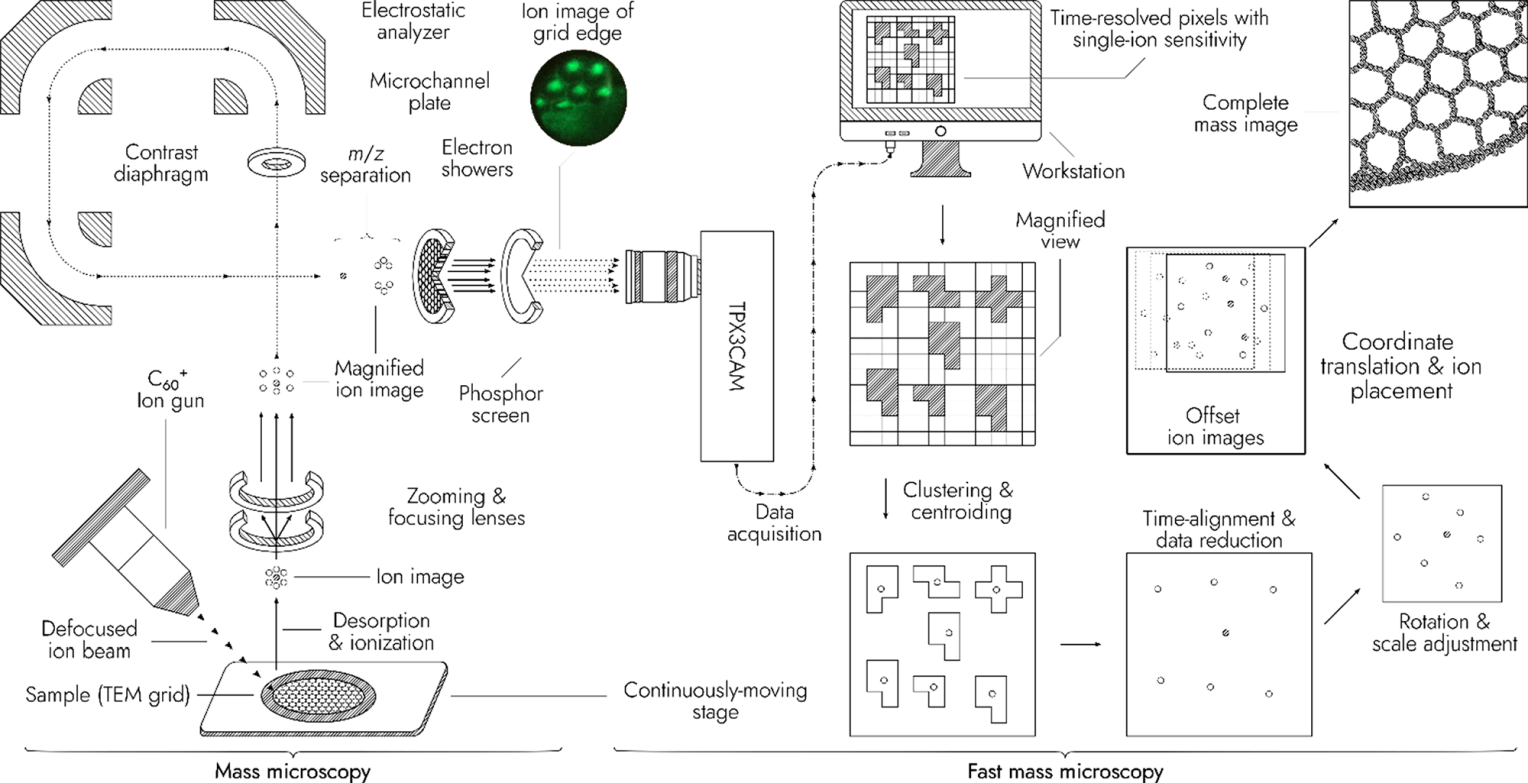
Scheme of the mass microscope modified with a TPX3CAM detector. A defocused C_60_^+^ ion beam irradiates a sample on a quickly and continuously moving stage, generating an ion image. The ion image is extracted into a TOF analyzer and magnified. The mass-separated ion image is projected onto a microchannel plate (MCP), producing electron showers. The electrons are converted to photons by a P20 phosphor screen. The photons are recorded with the TPX3CAM. The data are clustered and time-aligned to sets of single-ion impact coordinates. Ion coordinates are translated to sample stage coordinates, and a larger ion image of the stage-scanned sample surface is constructed. A photo of the phosphor screen (top-left corner) shows the distribution of surface ions when the fullerene ion beam irradiates the edge of a transmission electron microscopy (TEM) grid.

We would like to thank Andrei Nomerotski for useful discussions as well as Daniel Istrate and Henrique Pedrosa for performing XRF measurements to confirm the type of our phosphor screen as P20.

