# Supplementary material for: Fast Mass Microscopy: Mass Spectrometry Imaging of a Gigapixel Image in 34 Minutes
Source: Anal Chem. 2022 Oct 12;94(42):14652–8. doi: 10.1021/acs.analchem.2c02870 (PMC9607864; doi:10.1021/acs.analchem.2c02870)
Supplement: Supplementary file 1 — ac2c02870_si_001.pdf [file ac2c02870_si_001.pdf]

Supporting Information

Fast mass microscopy: mass spectrometry imaging of a gigapixel image in 34 minutes

Aljoscha Körber,[1] Joel D. Keelor,[2†] Britt S.R. Claes,[1] Ron M.A. Heeren[1]*, and Ian G.M. Anthony[1]

[1]The Maastricht MultiModal Molecular Imaging Institute (M4i), Division of Imaging Mass Spectrometry, Maastricht University, Universiteitssingel 50, 6229 ER Maastricht, the Netherlands

[2]Amsterdam Scientific Instruments B.V. (ASI), Science Park 106, 1098 XG Amsterdam, the Netherlands

Table of Contents

Conversion to .tpx3c file format S2

Conversion to .png and .izml file format S2

Mass calibration S2

Data and code availability S2

Supporting figures S3

S1 Photos showing the custom mounting bracket, with which the TPX3CAM and a TV zoom lens were mounted onto the stock detector unit of the BioTRIFT instrument. S3

S2 Imaging data of a 12.5 µm-pitch, copper TEM grid with 5 µm-wide bars, demonstrating a spatial resolving power of at least 5 µm and consistent with previous findings of at least 2.5 µm spatial resolving power. S4

S3 Comparing fast mass microscopy (left) with microprobe mode (right) at the example of TIC images of a 2.5 mm by 2.5 mm area of the cerebellum of the rat brain. S5

S4 Comparison of average mass spectra in microscope- and microprobe-mode MSI from the respective areas shown in Figure S3. .... S6

S5 Selected ion images of the microscope mode rat brain scan (seen in the main text as Figure 3). S7

S6 H&E-stained light microscopy and MSI images of serial mouse kidney sections. S8

S7 H&E-stained light microscopy and MSI images of serial human intestine sections. S9

S8 TIC MSI image of two overlaying TEM grids on an ITO slide acquired using fast mass microscopy.S10Supporting references S11

Conversion to .tpx3c file format

For conversion to the .tpx3c file format, the ion data in the .tpx3 file were (1) read by streaming, (2) clustered based on position and time information, (3) spatially centroided by weighing time-over-threshold (TOT) values, and (4) time-aligned to generate the individual ion hit information. The value used for time-alignment was the time-of-arrival (TOA) for all hits in the cluster. After time-alignment, the ion hit information was compressed by:

(1) converting each floating-point x and y TPX3CAM coordinate point to a set of nominal and a set of fractional (from values of 0/256 to 255/256) 8-bit unsigned integers (the nominal x and y 8-bit integers are because the TPX3CAM has 256×256 pixels; the fractional x and y 8-bit integers provide an additional 256 levels of inter-pixel accuracy),

(2) recording the number of activated pixels in the clustered hit,

(3) summing all activated pixels' TOTs (this summed TOT value provides a pseudo-intensity value that was not used for the purposes of this work, but was preserved in case it is useful in future works),

(4) generating a new, individual 8-byte pixel "packet" consistent with the .tpx3 file format and comprised of the x and y nominal coordinates, the time-aligned TOA, and the most significant bits of the summed TOT, and

(5) generating a second 8-byte "hit information packet" that contained the x and y fractional coordinate components, the clustered number of pixels, and the least significant bits of the summed TOT.

For each ion hit, these two, 8-byte packets were written to the new .tpx3c file. No compression was applied for clusters containing a single pixel. TPX3CAM time-to-digital (TDC) "packets" were stored in the new .tpx3c file. Generating the .tpx3c file accounted for approximately half of the elapsed real time for file conversion.

As .tpx3 files can be large and clustering and centroiding are computationally-intensive steps, conversion to the .tpx3c file format (developed for this project) serves as a method of lossy compression while also preserving clustering and centroiding information. The .tpx3c file format compresses each set of related pixels that are activated for a single ion hit. As such, if one or two pixels are activated, the .tpx3c file format does not offer any compression value. However, for hit events that activate 3 or more pixels, the filesize is reduced as if only 2 pixels were activated. Thus, if each ion hit each results in 4 activated pixels, the resulting .tpx3 file would be reduced in size by half by conversion to .tpx3c. If each ion hit resulted in 12 activated pixels, the resulting .tpx3c file would be (2 / 12) = 16.7% of the .tpx3c file size.

The .tpx3c file format is backwards-compatible with the .tpx3 file format as existing readers for .tpx3 file format can simply discard the 8-byte packets of "additional" summed TOT and fractional values as each 8-byte packet begins with a header. The header for a normal "hit" packet is 0xB (hexadecimal 11) whereas the packet for additional hit information is 0xC (hexadecimal 12).

Conversion to .png and .imzml file formats

The viewer of the data supplied the scan area width and height (in mm), the rotation of the TPX3CAM (in radians), the TPX3CAM field-of-view (in mm), x and y scaling factors, the size of the desired pixels, and a "peak time window" (or *m/z* window) that determines how wide the window around each mass peak will be for inclusion in a mass image for conversion to .png .imzml file formats. The mass window values were equal to the pulse width of the ion gun for the respective images. After these values were supplied, the .tpx3c file was read by streaming to determine the imaging row times, number of TDC events in each row, integrated mass spectrum, and location of any erroneously-active (i.e., "dead") TPX3CAM pixels. The imaging row times and number of time of flight (TOF) pulses in each row were used to calculate, via linear interpolation, a set of stage coordinates corresponding to each TDC event. The integrated mass spectrum was peak-picked to produce a mass list. The file was read by streaming either a second time (for .imzml conversion) or once for each peak in the mass list (for .png file conversion). Each hit was filtered by its TOF and whether it corresponded to a dead pixel. After passing the filter, each hit's x and y coordinates were translated from the TPX3CAM coordinate space to stage coordinate space by using the stage coordinates as offsets and applying rotation, stretch, and resizing operations. Once in stage coordinate space, each hit was then added to an in-memory buffer (with buffer size determined by the user-input pixel size) at the appropriate location. The addition of the hit incremented an intensity counter for the .png file. For the .imzml file, the addition of the hit recorded the *m/z* value. The buffer was then saved to a 16-bit, grayscale .png or an .imzml image file. For the .imzml file, the .ibd portion of the file was saved in "processed continuous" mode and used 32-bit floating point and 16-bit integers for the mass and intensity values, respectively.

Mass calibration

The TPX3CAM ion TOFs were converted to *m/z* values with a calibration function generated from known *m/z* peaks of indium tin oxide clusters, gold as well as of persistent contaminants, like sodium, potassium, and small organic fragments that were measured on ana clean ITO slide. TOF spectra were acquired with a 1 GHz digital oscilloscope (RTA4K-COM4, Rohde & Schwarz, Munich, DE) from the BioTRIFT microchannel plate (MCP), and the comparison measurements of the clean ITO slide and of the rat brain on the NanoTOF II.

Data and code availability

Data and code for the current study are available both on GitHub (https://github.com/M4i-Imaging-Mass-Spectrometry/fast-mass-microscopy-example) and on DataVerseNL (DOI: https://doi.org/10.34894/XKYD0Q).

▪ Supporting Figures

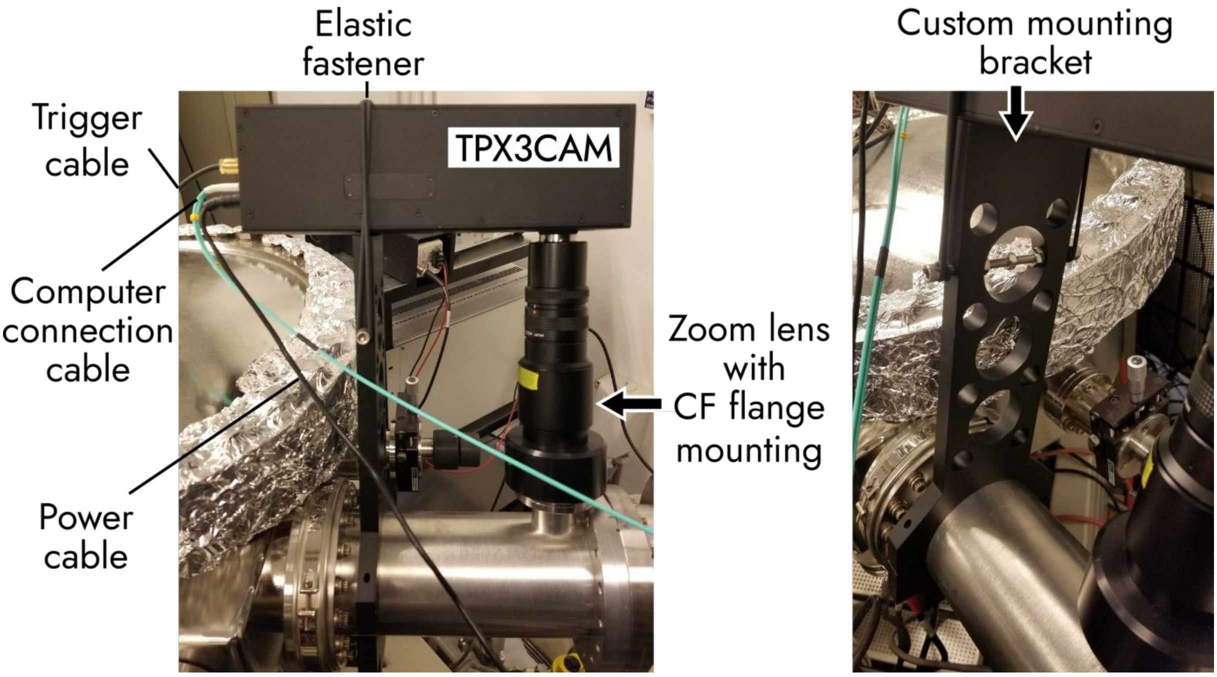

Supporting Figure 1. Photos showing the custom mounting bracket, with which the TPX3CAM and a TV zoom lens were mounted onto the stock detector unit of the BioTRIFT instrument. The stock detector unit inside the vacuum chamber consists of a micro-channel plate, a phosphor screen and a mirror, which guides photons, created at the phosphor plate, towards the TPX3CAM. The elastic fasteners allow for the TPX3CAM to be removed from the BioTRIFT instrument and used for other experiments on other instruments. This setup was easy to create and required minimal parts and no alterations of the BioTRIFT mass spectrometer or the stock TPX3CAM. The TPX3CAM simply takes the place of the stock, monochrome, charge-coupled-device (CCD) BioTRIFT camera.

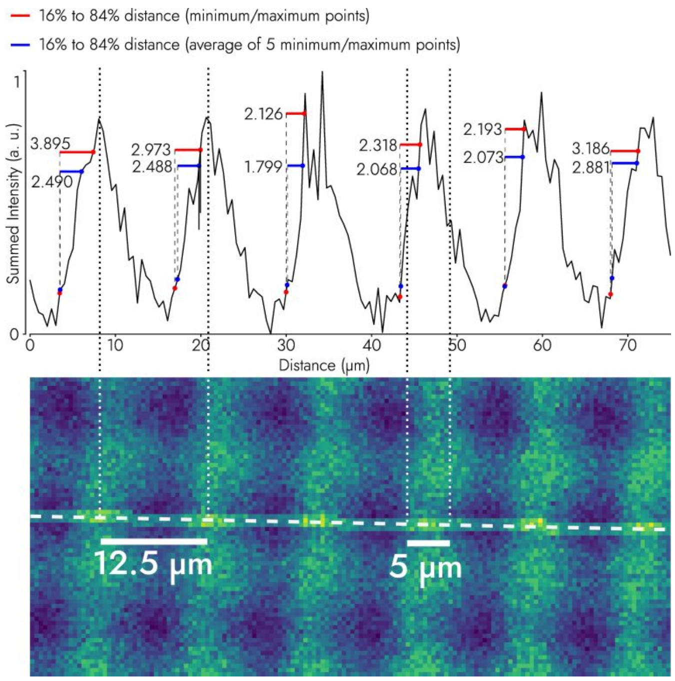

Supporting Figure 2. Imaging data of a 12.5 µm-pitch, copper TEM grid with 5 µm-wide bars, demonstrating a spatial resolving power on the grid edges of at least ≈2.5 µm, which is consistent with previous findings of at least 2.5 µm spatial resolving power.[1,2] Bottom: a portion of the TEM grid image constructed with a pixel size of 0.5 µm. A three-pixel-wide line, highlighted by brightening the pixels and overlaying a dashed, white line, was summed to produce the top graph. Top: a line-graph with peaks corresponding to centers of the TEM grid bars. Dotted lines were added to indicate corresponding points between the image and line graph. To estimate the minimum spatial resolving power we measured the average distance between pixels at 16% and 84% of the individual total peak intensity (highlighted by red horizontal bars; the average value is 2.78 µm with a standard deviation of 0.64 µm).[2] We also measured the average distance between groups of five pixels at 16% and 84% of the individual total peak intensity to account for noise in the data set (highlighted by blue horizontal bars; the average value is 2.30 µm with a standard deviation of 0.36 µm).These results are comparable to previous determinations on the same instrument in static-analysis mode made by Jungmann et al. and McDonnell et al. who reported values of at least 3.4 µm and 2.5 µm using MALDI and SIMS, respectively.[1,3] Proper resolution of a normally-distributed peak requires multiple data points, therefore we visualized **Figure S2** with 0.5 µm pixels. Previously, a lateral resolving power of 1 µm was reported using SIMS on a prototype TRIFT-based instrument.[4]

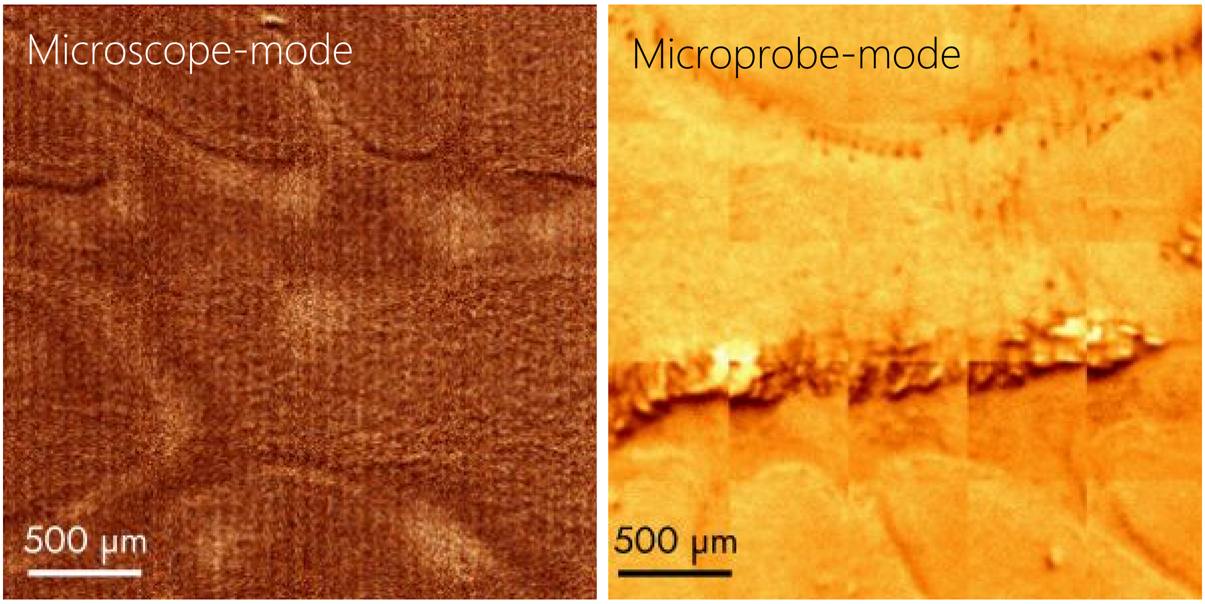

Supporting Figure 3. Comparing fast mass microscopy (left) with microprobe mode (right) at the example of TIC images of a 2.5 mm by 2.5 mm area of the cerebellum of the rat brain. The left image is a mirrored zoom-in of the cerebellum portion of **Figure 3**, displayed with 500 nm large pixels, and was acquired with the BioTRIFT instrument at an acquisition speed of 15,500 pixels s^{-1} using fast mass microscopy. The right image is of the same tissue area and was acquired with a NanoTOF II SIMS instrument in mosaic microprobe-mode with an acquisition speed of 200 pixels s^{-1} and a pixel size of 2 µm, which is the maximum spatial resolution achievable on the NanoTOF II instrument using a C_{60} ion gun. The ion sources for both images were identical model 20 kV C_{60}^{+} ion guns (Ionoptika, Chandler's Ford, UK). The total acquisition time of the right image amounted to 2 hours 11 minutes and 41 seconds, which we used as a benchmark for throughput in pulsed TOF-SIMS due to the lack of literature data. Acquiring the left side panel with four times higher spatial resolution than the right panel using fast mass microscopy required 26.5 minutes. Even though the brain was imaged in microscope-mode (**Figure 3** and the left panel) at a slower speed than the fingerprint image (**Figure 2**), the microscope-mode (left panel) acquisition rate is 80 times faster than microprobe-mode (right panel). Furthermore, measurements in microprobe-mode require focusing of the primary ion beam, which is time-consuming and can take up to several hours, especially at high spatial resolutions. Moreover, reaching an acquisition speed of 200 pixels s^{-1} in microprobe-mode produced several artefacts: (1) the raw images (*not shown*) contain clearly visible edges between tiles, (2) the upper right corner of every tile is undersampled, and (3) the tiles are misaligned to each other. These artefacts are the result of choosing 500 µm large tiles to maximize throughput in microprobe-mode to best compare the high-throughput capabilities of both modes. Therefore, primary ion beam aberrations occur at the edge of every tile. Choosing a greater number of smaller tiles will minimize these aberrations at the expense of reduced throughput. In this paper, we removed most of these artefacts after the measurement with a fast Fourier transform (FFT). Alternatively, there also are dedicated artefact removal algorithms for SIMS in the literature.[5] Other artefacts are a high-contrast "wrinkled" area between the second and third row of tiles, which is believed to be tissue damage that occurred when transferring the sample from the BioTRIFT to the NanoTOF II instrument, as well as vertical rows on the left image highlighting the edges of individual line scans. These rows can be avoided by choosing a higher oversampling ratio between line scans. The overall image contrast of the right image is inferior to fast mass microscopy. We attribute this to the overall smaller irradiation time per spot and to the visual impact of topological effects of microscope mode MSI (see **Figure S8**).

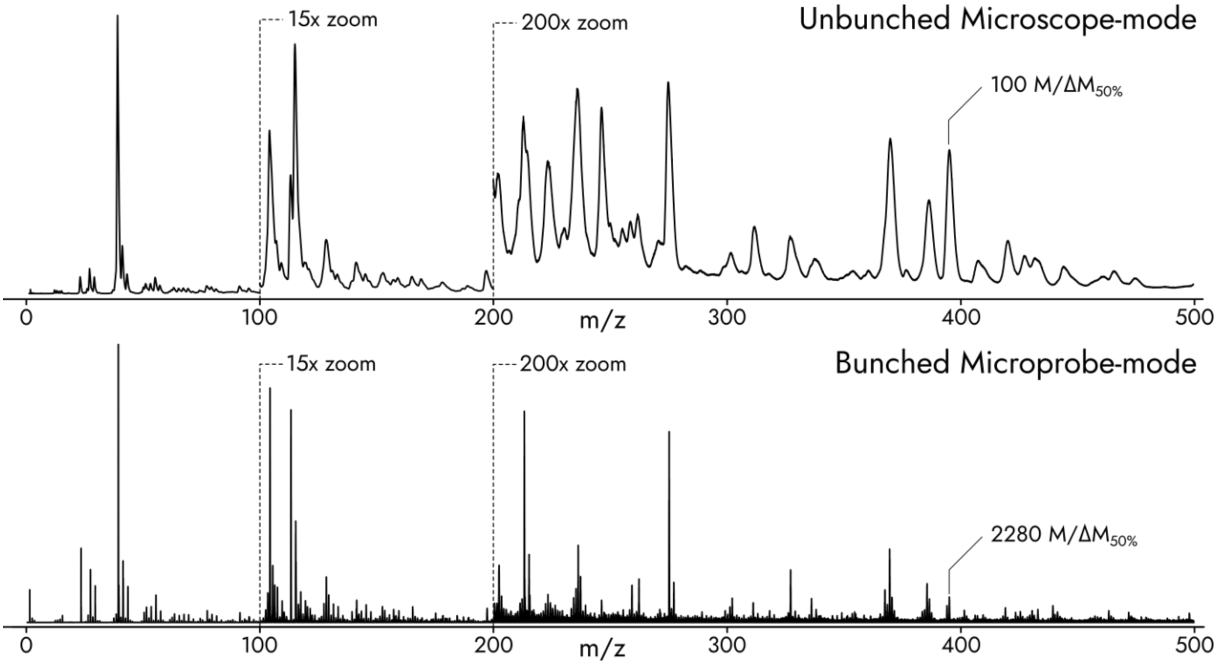

Supporting Figure 4. Comparison of average mass spectra in microscope- and microprobe-mode MSI from the respective areas shown in **Figure S3**. Both instruments yielded similar relative intensities. The TPX3CAM allows the observation of individual ion hits and its sensitivity is as high as MCP-based detection with microprobe-mode. Although similar in relative signal intensities, the mass resolution of the microscope-mode measurement is approximately 100 at m/z 395 whereas the mass resolution of the microprobe-mode acquisition is about 2,050 at m/z 395. This is caused by different primary ion pulse lengths as the C60 beam on the nanoTOF II instrument (microprobe-mode) was bunched while the ion beam on the microscope-mode setup was not bunched as that would have reduced the repetition rate and thus sensitivity of our setup. Other potential contributors are a long phosphor screen rise time as well as topology-induced peak broadening caused by slightly different starting positions of secondary ions on the sample surface.[6] In future measurements, we expect improvements in mass resolution of microscope-mode MSI by more than one order of magnitude by primary ion beam bunching, by the use of primary ion or laser pulses shorter than the 150 ns employed here, by using a phosphor screen with shorter rise time, and/or by topological deconvolution of the mass spectra.[6] Additionally, the development of the Timepix4 detector chip with a time resolution of 200 ps,[7] as well as the introduction of novel microscope-mode mass spectrometers[8,9] might enable reaching mass resolving powers up to 100,000.

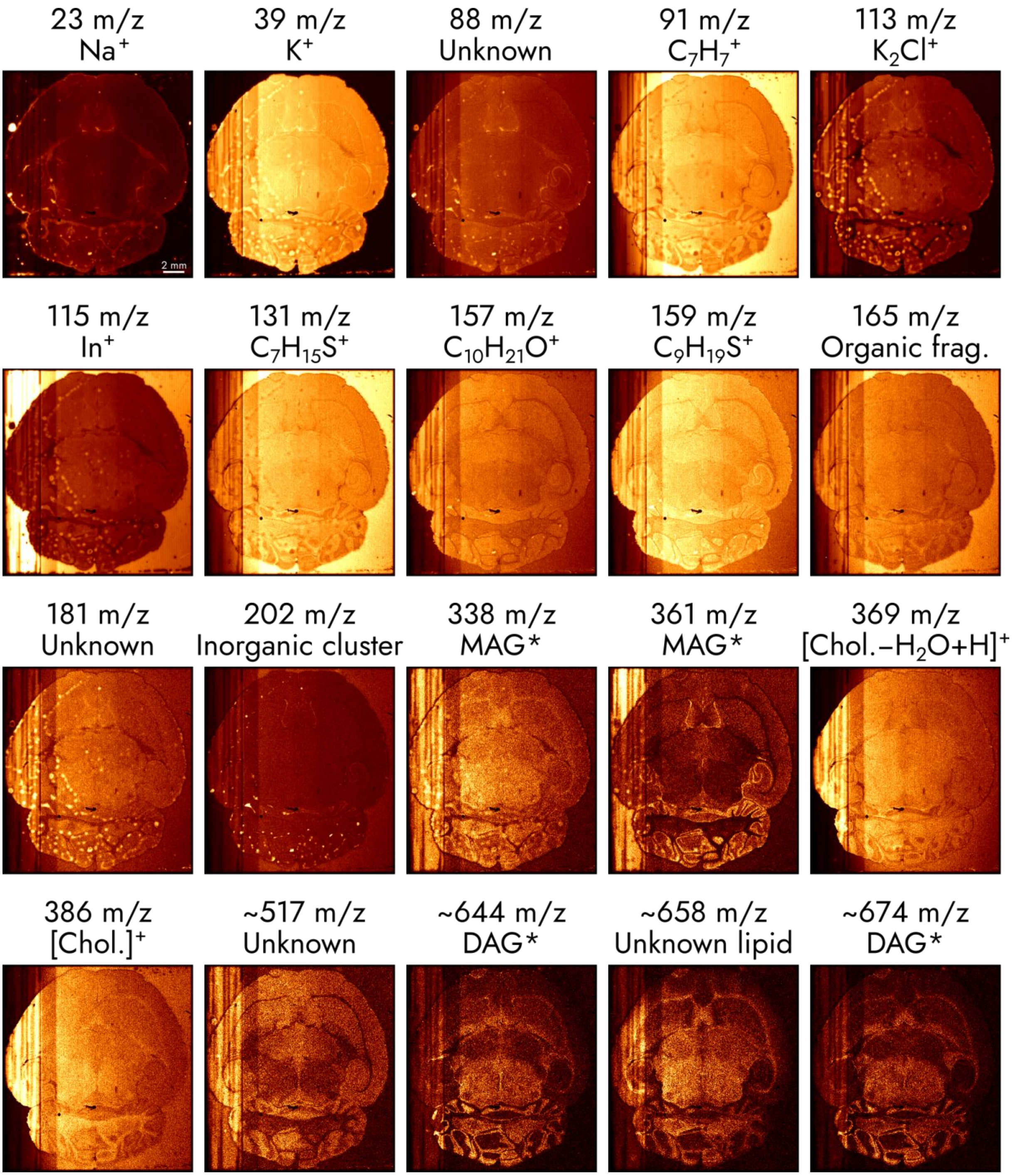

Supporting Figure 5. Selected ion images of the microscope mode rat brain scan (seen in the main text as **Figure 3**). The ion images are viewed at a pixel size of 50 µm to provide better contrast in the higher mass ion images that contain fewer ion hits than ion images at lower mass. This decrease in sensitivity towards larger molecules is intrinsic for SIMS in both, microprobe- and microscope mode.[10] Identifications, where possible, are provided below the *m/z* value.[11] Unambiguous assignments of some mass images require tandem MS and/or higher mass resolution and accuracy. Tentative assignments are marked with an asterisk(*). The abbreviations "Frag." and "Chol." stand for "fragment" and "cholesterol", respectively.

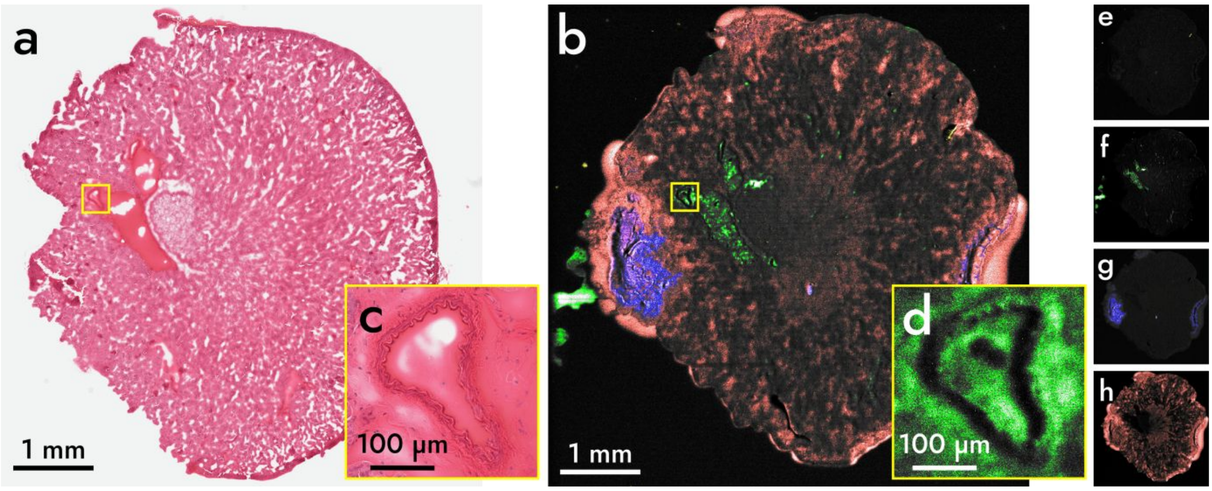

Supporting Figure 6. H&E-stained light microscopy and MSI images of serial mouse kidney sections. Light microscopy of hematoxylin and eosin (H&E)-stained tissue sections allows the visualization of cellular features at low cost and within the timeframe of a surgical procedure. It therefore has found widespread application in pathology for morphological assessment.[12] Acquired images are usually interpreted manually by a pathologist, which is time-consuming and the medical finding can vary between pathologists based on different levels of training and experience.[13,14] Thus, having access to a fast, chemically-sensitive method in addition to H&E-stained light microscopy has great benefit to pathologists. We therefore recorded an H&E-stained light microscopy image of a mouse kidney (a) and compared it with a false-color MSI image (b) of a consecutive tissue section acquired with fast mass microscopy at an acquisition speed of 15,500 pixels s^{-1} and with pixel sizes down to 500 nm. The imaging settings were identical to the settings used for the measurement of the rat brain in **Figure 3** of the main text. The mass image in (b) is shown with a pixel size of 5 µm and consists of four overlaid mass channels (*m/z* 165 (e), 39 (f), 86 (g), and 69 (h)), which are tentatively assigned to an unknown species, K^+, $C_5H_{12}N^+$, and $C_5H_9^+$, respectively. The yellow boxes in (a) and (b) delineate the magnified regions (c) and (d), respectively. The magnified mass image area (d) is viewed with a pixel size of 1 µm.

The H&E staining required 15 minutes, while MSI took 2:33:40. The H&E-stained image shows morphological features, such as the medulla, cortex, and blood vessels, at a high spatial resolution with pixel sizes of approximately 500 nm. The MSI image shows similar features as well as high molecular contrast in areas near the left, top, and right periphery of the tissue section. Furthermore, (d) exemplarily shows topological details.

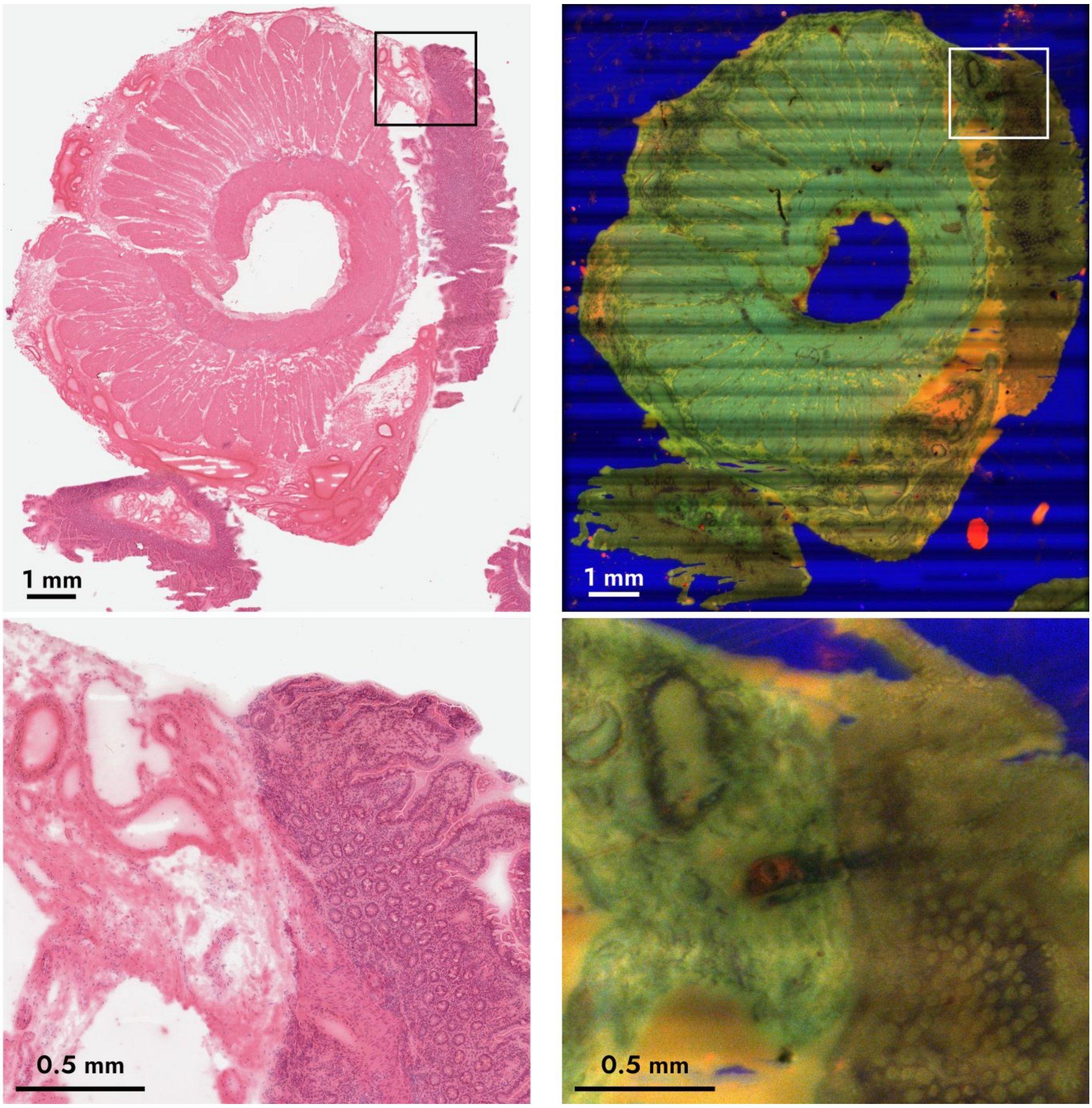

Supporting Figure 7. H&E-stained light microscopy (left) and false color MSI images (right; green color depicts K^+, red Na^+, and blue In^+) of serial human intestine sections (top), with corresponding zoom-ins demonstrate that fast mass microscopy on human tissue provides complementary chemical information to H&E stained light microscopy albeit with worse spatial resolving power. To improve readability of the mass image the zoom-in was slightly brightened. When viewed at 900 nm pixel size the image on the top right consists of 167 megapixels. The MSI image on the top right shows more of the stripe artefacts visible in Figures 3a and S5 attributed to an instability of an ion optic in the TRIFT II mass spectrometer causing variations in ion counts. Furthermore, the artefact of localized "bright" spots (Figure 3) does not occur here anymore due to minor optimization of our sample preparation workflow.

The H&E staining took 15 minutes while fast mass microscopy of the intestine tissue took 2:23:24 corresponding to approx. 19,400 pixels s^{-1} at 900 nm pixel size.

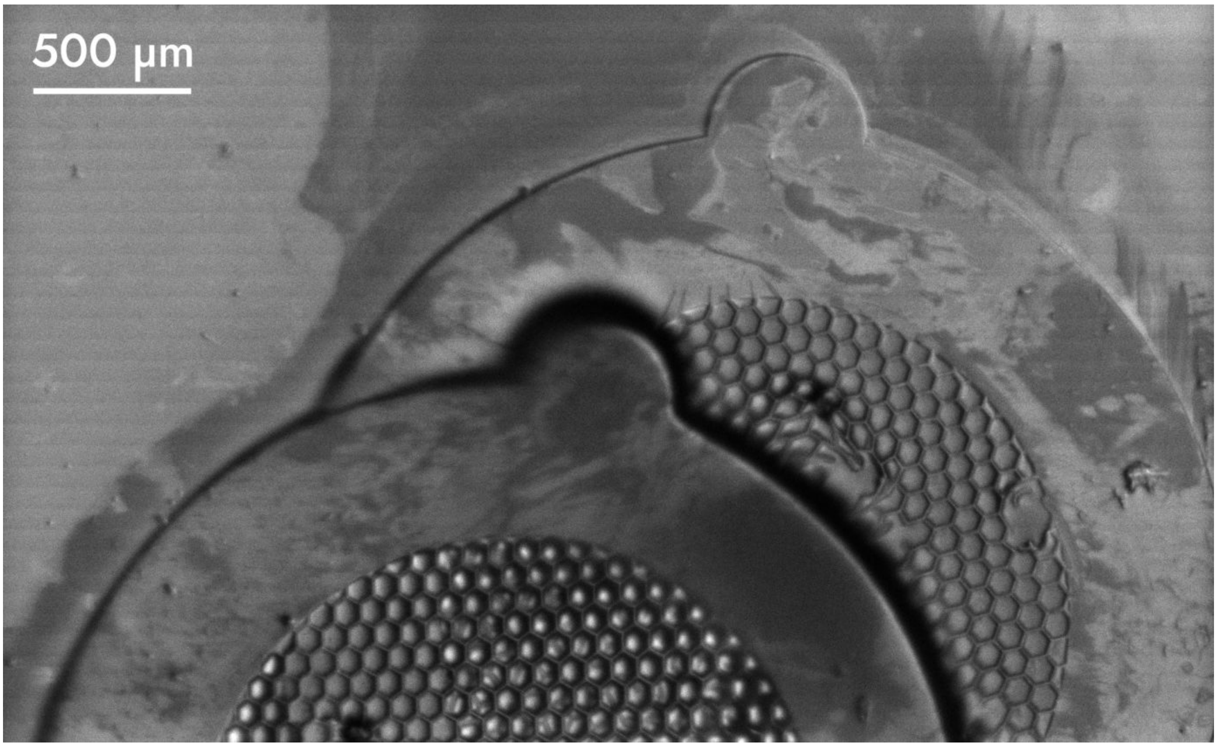

Supporting Figure 8. TIC MSI image of two overlaying TEM grids on an ITO slide acquired using fast mass microscopy. Microscope-mode MSI does not require a focused ion beam or laser, and thus allows for robust imaging of surfaces that vary in height. Height map profiles of these grids (not shown) were measured using a Keyence VHX-7000 microscope (Keyence, Osaka, Japan) and results showed that the distance between the highest point in this image (which is the top of the "tab" on the grid that is positioned on top of the other grid) and the ITO slide was at least 130 µm. The ability to image surfaces with "rough" topologies is advantageous as most biological tissues are not perfectly flat. This and the occurrence of shadow effects give access to topological information.

Supporting references

McDonnell, L. A.; Piersma, S. R.; Altelaar, A. F. M.; Mize, T. H.; Luxembourg, S. L.; Verhaert, P. D. E. M.; van Minnen, J.; Heeren, R. M. A. Subcellular Imaging Mass Spectrometry of Brain Tissue. *J. Mass Spectrom.* **2005**, *40* (2), 160–168. https://doi.org/10.1002/jms.735.

Dowsett, D.; Wirtz, T. Co-Registered in Situ Secondary Electron and Mass Spectral Imaging on the Helium Ion Microscope Demonstrated Using Lithium Titanate and Magnesium Oxide Nanoparticles. *Anal. Chem.* **2017**, *89* (17), 8957–8965. https://doi.org/10.1021/acs.analchem.7b01481.

Jungmann, J. H.; MacAleese, L.; Visser, J.; Vrakking, M. J. J.; Heeren, R. M. A. High Dynamic Range Bio-Molecular Ion Microscopy with the Timepix Detector. *Anal. Chem.* **2011**, *83* (20), 7888–7894. https://doi.org/10.1021/ac2017629.

Schueler, B.; Sander, P.; Reed, D. A. A Time-of-Flight Secondary Ion Microscope. *Vacuum* **1990**, *41* (7–9), 1661–1664. https://doi.org/10.1016/0042-207X(90)94047-T.

Gerber, F.; Marty, F.; Eijkel, G. B.; Basler, K.; Brunner, E.; Furrer, R.; Heeren, R. M. A. Multiorder Correction Algorithms to Remove Image Distortions from Mass Spectrometry Imaging Data Sets. *Anal. Chem.* **2013**, *85* (21), 10249–10254. https://doi.org/10.1021/ac402018e.

McDonnell, L. A.; Mize, T. H.; Luxembourg, S. L.; Koster, S.; Eijkel, G. B.; Verpoorte, E.; de Rooij, N. F.; Heeren, R. M. A. Using Matrix Peaks to Map Topography: Increased Mass Resolution and Enhanced Sensitivity in Chemical Imaging. *Anal. Chem.* **2003**, *75* (17), 4373–4381. https://doi.org/10.1021/ac034401j.

Llopart, X.; Alozy, J.; Ballabriga, R.; Campbell, M.; Casanova, R.; Gromov, V.; Heijne, E. H. M.; Poikela, T.; Santin, E.; Sriskaran, V.; Tlustos, L.; Vitkovskiy, A. Timepix4, a Large Area Pixel Detector Readout Chip Which Can Be Tiled on 4 Sides Providing Sub-200 Ps Timestamp Binning. *J.Instrum.* **2022**, *17* (01), C01044. https://doi.org/10.1088/1748-0221/17/01/C01044.

Hazama, H.; Aoki, J.; Nagao, H.; Suzuki, R.; Tashima, T.; Fujii, K. ichi; Masuda, K.; Awazu, K.; Toyoda, M.; Naito, Y. Construction of a Novel Stigmatic MALDI Imaging Mass Spectrometer. *Appl. Surf. Sci.* **2008**, *255* (4), 1257–1263. https://doi.org/10.1016/j.apsusc.2008.05.058.

Verenchikov, A. N.; Yavor, M. I. Imaging Properties of a Multi-Reflection Time-of-Flight Mass Analyzer. *Int. J. Mass Spectrom.* **2021**, *463*, 116547. https://doi.org/10.1016/j.ijms.2021.116547.

McDonnell, L. A.; Heeren, R. M. A. Imaging Mass Spectrometry. *Mass Spectrom. Rev.* **2007**, *26* (4), 606–643. https://doi.org/10.1002/mas.20124.

Gross, J. H. *Mass Spectrometry*, 3rd ed.; Springer International Publishing: Cham, 2017. https://doi.org/10.1007/978-3-319-54398-7.

Rosai, J. Why Microscopy Will Remain a Cornerstone of Surgical Pathology. *Lab. Investig.* **2007**, *87* (5), 403–408. https://doi.org/10.1038/labinvest.3700551.

El-Badry, A. M.; Breitenstein, S.; Jochum, W.; Washington, K.; Paradis, V.; Rubbia-Brandt, L.; Puhan, M. A.; Slankamenac, K.; Graf, R.; Clavien, P. A. Assessment of Hepatic Steatosis by Expert Pathologists: The End of a Gold Standard. *Ann. Surg.* **2009**, *250* (5), 691–696. https://doi.org/10.1097/SLA.0b013e3181bcd6dd.

Elmore, J. G.; Longton, G. M.; Carney, P. A.; Geller, B. M.; Onega, T.; Tosteson, A. N. A.; Nelson, H. D.; Pepe, M. S.; Allison, K. H.; Schnitt, S. J.; O'Malley, F. P.; Weaver, D. L. Diagnostic Concordance among Pathologists Interpreting Breast Biopsy Specimens. *JAMA – J. Am. Med. Assoc.* **2015**, *313* (11), 1122–1132. https://doi.org/10.1001/jama.2015.1405.
